# Assessment of Point of Care Lung Ultrasound in the Ambulatory Heart Failure Setting

**DOI:** 10.1002/clc.70244

**Published:** 2026-02-11

**Authors:** David Golombeck, Radiah Khandokar, Joanna Fishbein, Allison Provenzale, Melodie Lin, Marsha McGee, Dora Rossi, Simon Maybaum

**Affiliations:** ^1^ Cardiovascular Institute, Northwell New Hyde Park New York USA; ^2^ Biostatistics Unit Office of Academic Affairs, Northwell New Hyde Park New York USA

## Abstract

**Background:**

Limited studies have evaluated lung ultrasound (LUS) in ambulatory heart failure (HF). A six‐zone LUS assesses B‐lines, a marker of congestion. The Butterfly IQ+ probe features an automated B‐line counter (ABLC), eliminating manual counting. We evaluated LUS quality by novice HF providers after training, compared expert manual counts to ABLC, and explored associations between LUS and clinical HF metrics.

**Methods:**

Three novice providers underwent 2 h of didactics and 30 proctored exams. Image quality was independently reviewed by two LUS experts. B‐lines were counted manually by experts and ABLC. We assessed associations between LUS and four clinical metrics: provider‐assessed volume status, > 30% NT‐proBNP increase, > 5 lb weight gain, and PAD above goal (CardioMEMS).

**Results:**

Seventy‐five subjects were enrolled. Overall, LUS quality was excellent, with 88% good quality. Surprisingly, agreement between expert B‐line counts was moderate (Gwet's AC1: 0.49, 95% CI: 0.27 to 0.71) while the accuracy of experts as compared to ABLC was modest (Expert 1 = 61.2%, Expert 2 = 40.3%). Experts correctly identified 93% of positive studies but only 19% of negative studies versus ABLC. Provider volume assessments substantially agreed with LUS (Gwet's AC1: 0.76, 95% CI: 0.61 to 0.91), but providers identified only half of positive LUS cases, suggesting utility in detecting mild volume overload. Only volume overload correlated with positive LUS. Only 25% of subjects had a CardioMEMS.

**Conclusion:**

Novice providers can perform high‐quality LUS after brief training. ABLC reduces B‐line counting variability. LUS detects mild pulmonary congestion undetectable by clinical exam, potentially preventing worsening in HF patients.

## 1 Introduction

**Figure 1:**
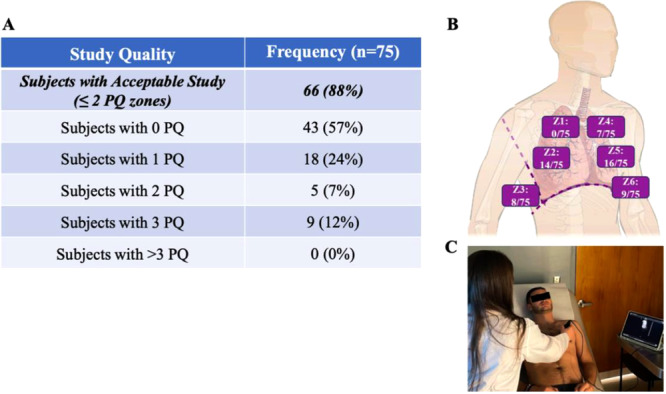
LUS quality analysis. (A) Number of PQ zones by subject. (B) Distribution of PQ zones by location. (C) Performance of LUS examination. GQ = good quality, LUS = lung ultrasound, PQ = poor quality.

Readmission rates for patients with heart failure (HF) after hospital admission remain high, and close surveillance in the outpatient setting is key to improving outcomes. Specifically, detecting volume overload has been shown to prevent readmission and improve outcomes in HF [1]. Multiple studies in the inpatient setting have demonstrated the value of point‐of‐care (POC) lung ultrasound (LUS) in the management of HF through the detection of pulmonary congestion [2, 3]. In a recent multicenter study utilizing POC LUS in over 400 subjects, B‐line count was associated with NT‐proBNP levels and key echocardiographic findings [4]. A six‐zone LUS examination (Figure 1B) has been utilized to assess B‐lines [4], a measure of congestion. Prior studies have demonstrated an association between B‐lines and NT‐pro‐BNP [5, 6], chest X‐ray [6], and physical examination [5, 6, 7].

Limited prior studies have evaluated LUS in HF patients in the ambulatory setting, and only one has utilized LUS performed by novice providers after a period of training [7]. In that study, the quality of LUS after training was not specifically assessed.

While different methodologies exist for assessing B‐line count, ≥ 3 total B‐lines across all zones have been utilized in the HF ambulatory setting to guide care [5, 7]. The Butterfly IQ+ (Guilford, CT) is a handheld, commercially available LUS probe system that can interface with a smartphone or tablet, offering convenient POC assessment in the ambulatory setting. This device has a novel automated B‐line counter (ABLC), avoiding the need for manual counting by the provider.

The primary goal of this study was to evaluate the quality of LUS performed by novice providers in the HF clinic after a formal period of training. Additionally, we assessed the reliability of manual B‐line counting by LUS experts as compared to ABLC, and finally, we explored the association between positive LUS and clinical measures of HF.

## 2 Methods

### 2.1 Patient Population

This was a prospective, single‐center, observational study conducted at the outpatient HF program at Northshore University Hospital. Seventy‐five subjects with any stage of HF (HFpEF or HFrEF) were enrolled between July and November 2023. Only subjects with severe lung disease were excluded. This protocol was approved by the institutional IRB, and written informed consent was obtained from all subjects. The investigation conforms with the principles outlined in the *Declaration of Helsinki*.

### 2.2 Provider LUS Training

Three HF nurse practitioners (NP) with no prior LUS experience underwent 2 h of didactics, from the online Butterfly education portal, followed by at least 30 in‐person proctored examinations with the Butterfly IQ+, in accordance with industry standards for LUS training [8].

### 2.3 Study Visit and LUS Acquisition

All study procedures were performed during one routine clinic visit. NPs were asked to designate subjects as euvolemic or volume overload based upon clinical examination. The following additional three HF clinical measures were assessed: pulmonary artery diastolic pressure (PAD) above goal (subjects with CardioMems), > 20% increase over the lowest NT‐proBNP in the prior 12 months, and ≥ 5 lb weight gain over the prior 3 months.

At the end of the clinic visit, a six‐zone LUS was performed (Figure 1B,C) by the provider using the Butterfly IQ+. Images were uploaded to the cloud, and offline image assessment for quality and B‐lines was performed by two external LUS experts who were blinded to the clinical data.

### 2.4 Image Quality Analysis

Three expert clinicians were identified who had undergone LUS instruction as a core competency for emergency medicine training and had each interpreted over 1000 studies. Experts scored each zone for quality utilizing the American College of Emergency Physicians (ACEP) Quality Assurance 5‐point Grading Scale [8]. Zones were considered good quality (GQ) with an ACEP score ≥ 3 and poor quality (PQ) with a score < 3. A third expert adjudicated in the event of disagreement between experts. A LUS was considered acceptable if there were ≤ 2 PQ zones. Quality was reported by zone and for the study in its entirety.

### 2.5 B‐Line Analysis

For each LUS study, B‐lines were counted by the two experts and by the Butterfly ABLC. Subjects were considered to have a positive LUS if there were ≥ 3 B‐lines across all six zones [5, 7]. Subjects with a positive LUS by ABLC were included in the B‐line analysis, irrespective of the number of PQ zones. Subjects with a negative study by ABLC and > 2 PQ zones were excluded from B‐line analyses due to PQ. We analyzed the agreement between experts and the agreement between experts and the ABLC as to whether a study was positive or negative. The accuracy of the experts was reported as the proportion of true results, either true positive or true negative. We further assessed associations between LUS and the four HF measures.

### 2.6 Statistical Analyses

For all analyses, descriptive statistics were computed (e.g., frequencies and proportions for categorical data and means and standard deviations or medians, first quartile to third quartile for continuous data) as appropriate. LUS quality was reported using descriptive statistics at both the individual zone and subject level. The quality of dependent zones (Z3 and Z6) were compared to non‐dependent zones (Z1−Z2, Z4−Z5) utilizing a Chi‐square test. The accuracy of each individual expert with respect to B‐line count (as compared to ABLC) was reported along with the corresponding 95% exact binomial confidence interval. Agreement between experts and ABLC with respect to B‐line count and between providers and LUS with respect to volume status was assessed using Gwet's Agreement Coefficient (AC1) [9]. Tests of association between positive or negative LUS and HF measures were performed using Fisher's exact tests. Results yielding a *p* < 0.05 were considered statistically significant. All analyses were conducted using SAS version 9.4 (SAS Institute Inc., Cary, NC).

## 3 Results

### 3.1 Study Population

Baseline characteristics of the study cohort are shown in Table 1. On average, subjects were 69 ± 13 years old, 76% were male, 61% had HFrEF, and over 90% were NYHA Class II and above. Average BMI was 28 ± 6, reflecting a predominantly overweight to obese population. Eleven subjects (15%) had a recent HF hospitalization (<30 days) prior to the study. Only 58% of subjects were on standing daily diuretics, reflecting a predominantly stable ambulatory population. Of subjects with HFrEF, 70% were on at least three HF medications, and 55% of subjects with HFpEF were managed with an SGLT2 inhibitor and/or an MRA.

**Table 1:** Baseline characteristics (*n* = 75).

Age (years; mean ± SD)	69 ± 13
Male Sex, *n* (%)	57 (76)
BMI (kg/m²; mean ± SD)	28 ± 6
HF Hospitalizations within 30 days, *n* (%)	11 (15)
Ischemic, *n* (%)	47 (63)
NYHA Class I/II/III, *n* (%)	7 (9)/52 (69)/16 (22)
HFrEF, *n* (%)	46 (61)
Medications	
Beta‐blocker, *n* (%)	64 (85)
ACE‐I/ARB/ARNI, *n* (%)	49 (65)
MRA, *n* (%)	41 (63)
SGLT2 Inhibitor, *n* (%)	47 (63)
Daily Diuretics, *n* (%)	38 (58)
CardioMems, *n* (%)	19 (25)
NT‐pro BNP (pg/mL; Median [IQR])	287 (1471)

Abbreviations: ACE‐I, angiotensin‐converting enzyme inhibitor, ARB, angiotensin receptor blocker, ARNI, angiotensin receptor/neprilysin inhibitor; BMI, body mass index (mean ± SD); NT‐pro BNP, N terminal pro‐b type natriuretic peptide (median [IQR]); mineralocorticoid receptor antagonist; SGLT2, sodium‐glucose cotransporter 2.

### 3.2 Quality Analysis

A total of 450 zones were imaged in 75 subjects, and an adjudicator for quality was required in 64/450 (14%) zones. Figure 1A shows the number of PQ zones by subject, and Figure 1B shows the distribution of PQ zones by location. Overall, 66 (88%) subjects had acceptable LUS study for quality, and no subjects had more than 3 PQ zones. There was no significant difference in the proportion of PQ zones in the dependent (Z3, Z6) vs non‐dependent zones (11% vs. 12%, *p* = 0.76). There was also no significant difference in the proportion of poor‐quality studies between those with BMI less than or greater than 30.

### 3.3 B‐Line Analysis

Eight subjects with PQ studies (> 2 PQ zones) and a negative study by ABLC were excluded from the B‐line analysis. However, one subject with > 2 PQ zones and a positive study by ABLC was included, leaving 67 subjects included in the B‐line analysis cohort.

***Concordance of Expert's Manual B‐line Counts***. Both experts performed manual B‐line counting on each study, and each expert defined a study as positive (≥ 3 B‐lines) or negative. Surprisingly, we found only moderate agreement between the two experts' interpretations as to whether a study was positive or negative (Gwet's AC1: 0.49, 95% CI: 0.27 to 0.71).

**Figure 2:**
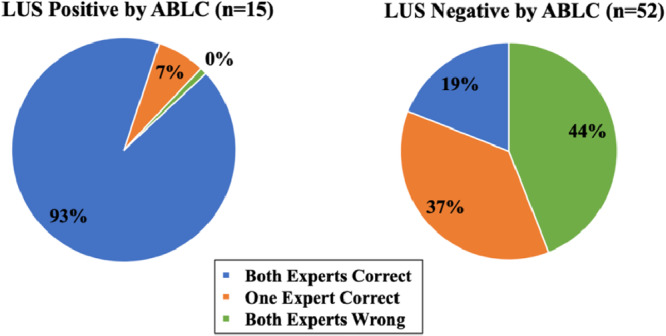
B‐line analysis: Expert Manual Count versus ABLC. Accuracy of the experts' manual B‐line count compared with the ABLC. Both experts were correct 93% of the time when the study was positive, but only 19% of the time when the study was negative. ABLC = Automated B‐line Counter; LUS = Lung Ultrasound.

***Accuracy of Experts' Manual B‐line Count versus ABLC***. We defined a study as positive if there were ≥ 3 B‐lines by ABLC. Both experts were correct 93%of the time when the study was positive, but only 19%of the time when the study was negative (Figure 2). Of note, when the study was positive, at least one expert was correct all the time, and when the study was negative, at least one expert was correct 56% of the time.

Overall accuracy of the individual experts was modest (Expert 1 = 61.2%, 95% CI: 48.5% to 72.9%; Expert 2 = 40.3%, 95% CI: 28.5% to 53.0%). It should be noted that accuracy should be interpreted based upon the observed prevalence of 22.4% of positive LUS in our study population, which is consistent with prior studies in the outpatient setting [5, 7].

**Figure 3:**
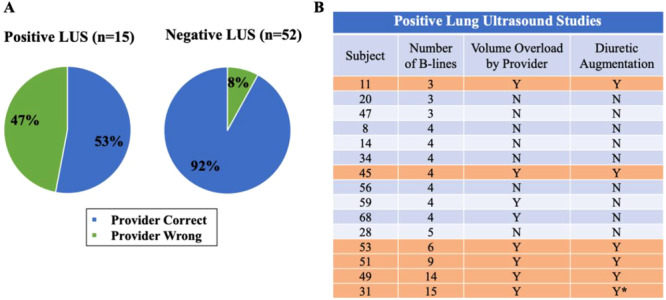
Provider assessment of volume status versus LUS. (A) Agreement of provider assessment of volume status with LUS. Providers' assessments were correct 92% of the time when the study was negative, but they only identified volume overload in half of the subjects with a positive LUS. B. Table showing details of the 15 subjects with positive LUS by ABLC. Number of B‐lines, provider assessment of volume status, and whether diuretics were augmented are shown. Orange‐shaded rows indicate diuretic augmentation. *Denotes the only subject admitted to hospital for intra‐venous diuretics. All subjects who had six or more B‐lines required diuretic augmentation; only the subject with the highest B‐line count required admission for intra‐venous diuretics. ABLC = automated B‐line counter, LUS = lung ultrasound, N = no, Y = yes.

***LUS Results***. Overall, only 22% (15/67) of the LUS were positive (≥ 3 B‐lines by ABLC). The median number of total B‐lines per subject was 0.5 (IQR 0–2). Among positive studies, the median B‐line count was 4 (range 3−15) (Figure 3B). Only 3 subjects had ≥ 9 B‐lines.

***Agreement of Provider Clinical Assessment and LUS*** (Figure 3). Overall, the agreement between the provider assessment of volume status and LUS was substantial (Gwet's AC1: 0.76, 95% CI: 0.61 to 0.91) with the 95% confidence level indicating a level of agreement ranging from substantial to almost perfect. Providers were correct 92% of the time when the study was negative. In contrast provider's clinical examination only identified half (53%) of the 15 subjects with pulmonary congestion by LUS, suggesting that LUS may have been helpful in these subjects with mild volume overload. Importantly, all the subjects who had six or more B‐lines required diuretic augmentation, and only the subject with the highest B‐line count (15 B‐lines) required admission to the hospital for intra‐venous diuretics. It should be noted that the clinical examination was performed prior to the LUS and that the providers did not know the B‐line count at the time of the encounter, thus not influencing their clinical decision‐making.

***Association Between LUS and Clinical HF Measures*** (Table 2). Tests of association between positive or negative LUS and the four HF measures were performed and shown in Table 2. A significantly higher proportion of subjects with volume overload had a positive LUS than a negative LUS (53% vs. 8%, *p* = 0.0003). Individually, neither BNP, PAD, nor weight gain was significantly associated with a positive LUS. However, subjects with at least one positive HF measure were significantly more likely to have a positive than a negative LUS (80% vs. 37%, *p* = 0.0037).

**Table 2:** Association between LUS and HF metrics.

	Positive LUS (*n* = 15)	Negative LUS (*n* = 52)	*p* value
Volume Overload by Clinical Exam (*n* = 12) Total included subjects (*n* = 67)	8 (53%)	4 (8%)	**0.0003**
Elevated BNP (*n* = 15) Total included subjects (*n* = 48)	5 (50%)	10 (26%)	0.25
PAD Above Goal (*n* = 5) Total included subjects (*n* = 18)	3 (37.5%)	2 (20%)	0.61
Weight Gain (*n* = 16) Total included subjects (*n* = 55)	5 (33%)	11 (24%)	0.30
Any HF metric (*n* = 31) Total included subjects (*n* = 67)	12 (80%)	19 (37%)	**0.0037**

*Note:* Columns are not mutually exclusive.

Abbreviations: BNP, brain natriuretic peptide; HF, heart failure; LUS, lung ultrasound; PAD, pulmonary artery diastolic pressure.

## 4 Discussion

In this study, we found that after a brief period of training, providers were able to obtain high‐quality LUS images, with 88% of subjects having good‐quality studies. We further showed only moderate agreement between two independent experts performing manual B‐line counts and only modest accuracy in determining if a study was positive or negative. Providers were almost always correct in identifying subjects without volume overload; however, they only identified half the subjects with a positive LUS as having volume overload. In an exploratory analysis of clinical measures of HF, only provider assessment of volume overload was significantly associated with a positive LUS.

Only 22% of subjects had a positive LUS, consistent with prior studies in the outpatient setting [5, 7]. Of note, the median B‐line count in this study was lower than that reported in a recent multi‐center LUS study in ambulatory HF patients [4](1 vs. 3). Almost all other studies utilizing LUS in HF have been conducted in the inpatient setting, with positive LUS ranging from 47% to 65% [10, 11, 12]. Lower prevalence of positive LUS in the ambulatory population likely impacts both the accuracy of the expert's B‐line assessment and the accuracy of the provider assessment of volume status.

Our threshold for a positive LUS was ≥ 3 total B‐lines in accordance with prior literature in the ambulatory setting [5, 7]. Inpatient studies have utilized diverse B‐line thresholds to define a positive LUS [10, 11], with generally higher thresholds (4‐15 B‐lines) than those used in the ambulatory setting. In our study, providers were almost always correct in identifying subjects without volume overload who had a negative LUS, however, they only identified half the subjects with a positive LUS as having volume overload. The low B‐line threshold we utilized may reflect mild volume overload, not always detected by clinical exam. One prior study in the ambulatory setting has suggested that even mild pulmonary congestion by LUS is associated with worse outcomes [5]. Whether early identification of mild pulmonary congestion by LUS is helpful in preventing clinical worsening should be the focus of future studies.

We found only moderate agreement between two experts performing manual B‐line counts (as to whether LUS was positive or negative), supporting the value of the ABLC as a standardization tool. Notably, both the experts' manual count overclassified LUS as positive 48% and 77% of the time, respectively.

The analysis of the association between LUS and some of the clinical HF measures is limited by small sample size due to missing data (specifically for CardioMems and BNP, Table 2), and we view these analyses as exploratory. Further, we did not assess the relationship between positive LUS and future clinical events. It should be noted that only 25% of patients had a CardioMems device, which is a limitation to any association between PAD and B‐line count.

In conclusion, providers with no prior LUS experience can perform good‐quality pulmonary scans in the ambulatory HF setting after a brief period of training. Use of an ABLC simplifies the process and diminishes variability of interpretation. Future studies are needed to assess whether early identification of mild pulmonary congestion by LUS (not detectable by clinical examination) may prevent clinical worsening.

## Author Contributions

Drs. Simon Maybaum and David Golombeck conceived and designed the study and provided overall supervision of the project. Dr. Golombeck and Ms. Radiah Khandokar contributed to data collection, manuscript writing, and editing. Ms. Johanna Fishbein conducted the statistical analyses and contributed to manuscript review and editing. Allison Provenzale, Melodie Lin, Marsha McGee, and Dora Rossi assisted with data collection. All authors reviewed and approved the final manuscript and agree to be accountable for all aspects of the work.

## Conflicts of Interest

A research grant from Butterfly Inc was received. Butterfly Inc played no role in the design, execution, data analysis, or manuscript preparation. Butterfly Inc was given an opportunity to review the aggregate data and give comments on the manuscript, however, the content of the manuscript was solely at the discretion of the authors. The other authors declare no conflicts of interest.

## Data Availability

The data that support the findings of this study are available on request from the corresponding author. The data are not publicly available due to privacy or ethical restrictions.
